# Methylation of *MGMT* Is Associated with Poor Prognosis in Patients with Stage III Duodenal Adenocarcinoma

**DOI:** 10.1371/journal.pone.0162929

**Published:** 2016-09-19

**Authors:** Tao Fu, Anup Sharmab, Fei Xie, Yanliang Liu, Kai Li, Weiwei Wan, Stephen B. Baylin, Christopher L. Wolfgang, Nita Ahuja

**Affiliations:** 1 Department of Gastrointestinal Surgery II, Key Laboratory of Hubei Province for Digestive System Disease, Renmin Hospital, Wuhan University, Wuhan, China; 2 Department of Surgery, The Johns Hopkins University School of Medicine, Baltimore, Maryland, United States of America; 3 Department of Oncology, The Johns Hopkins University School of Medicine, Baltimore, Maryland, United States of America; 4 Department of Urology, The Johns Hopkins University School of Medicine, Baltimore, Maryland, United States of America; Beijing Cancer Hospital, CHINA

## Abstract

**Background:**

*O*^*6*^*-methylguanine-DNA methyltransferase* (*MGMT*) methylation status has not been extensively investigated in duodenal adenocarcinoma (DA). The aim of this study was to evaluate the *MGMT* methylation status and examine its possible prognostic value in patients with stage III DA.

**Methods:**

Demographics, tumor characteristics and survival were available for 64 patients with stage III DA. *MGMT* methylation was detected by using MethyLight. A Cox proportional hazard model was built to predict survival, adjusted for clinicopathological characteristics and tumor molecular features, including the CpG island methylator phenotype (CIMP), microsatellite instability (MSI), and *KRAS* mutations.

**Results:**

*MGMT* methylation was detected in 17 of 64 (26.6%) patients, and was not correlated with sex, age, tumor differentiation, CIMP, MSI, or *KRAS* mutations. *MGMT* methylation was the only one factor associated with both overall survival (OS) and disease-free survival (DFS) on both univariate and multivariate analyses. In patients treated with surgery alone, *MGMT*-methylated group had worse OS and DFS when compared with *MGMT*-unmethylated group. However, in patients treated with chemotherapy/radiotherapy, outcomes became comparable between the two groups.

**Conclusions:**

Our results demonstrate *MGMT* methylation is a reliable and independent prognostic factor in DAs. Methylation of *MGMT* is associated with poor prognosis in patients with stage III DAs.

## Introduction

Primary adenocarcinoma of the duodenum (duodenal adenocarcinoma, DA) was initially described by Hamburger in 1746, comprising less than 1% of all malignant neoplasms of the gastrointestinal tract [1–3]. Because of its rarity, there is an insufficiency of well-designed studies to guide management. In general, DAs have more favorable outcomes compared to other periampullary malignancies and excision is considered the backbone of treatment for patients with localized tumors or limited metastatic disease when feasible. Data regarding the effect of adjuvant chemotherapy/radiotherapy are limited, with no faithful evidence of significant benefit in survival in patients with DAs. A Cochrane review in 2007 failed to find suitable trials eligible for meta-analysis to determine the role of adjuvant chemotherapy in the treatment of adenocarcinoma of the small intestine [4]. Although adjuvant therapy is regularly used in this disease, more studies are needed to evaluate the effectiveness of adjuvant therapy in the management of DAs.

O^6^-methylguanine-DNA methyltransferase (MGMT) is a ubiquitously expressed DNA repair protein, and it removes methyl and chloroethyl groups from the O^6^ position of guanine in a damage reversal reaction. In the absence of MGMT, O^6^-methylguanine in the DNA generates point mutations and DNA double-strand breaks via cellular replication and DNA mismatch repair that trigger cell death by apoptosis [5]. Methylation of the CpG islands located in the promoter region of *MGMT* is primarily responsible for the inactivation of MGMT in several tumor types [6]. Inactivation of MGMT can lead to it subsequently being unable to protect tumors from cytotoxic damage induced by alkylating chemotherapeutics, i.e. methylating and chloroethylating agents, and thus predicts benefit from these chemotheraptic agents. *MGMT* methylation may also play a prognostic role in various cancers. To our knowledge, there is only one previous study that has described *MGMT* methylation in DAs in a small number of patients and there was no assessment of *MGMT* methylation frequency or prognostic significance [7].

Microsatellite instability (MSI), developing from defects in other mismatch repair genes *MLH1*, *MSH2*, *MSH6*, and *PMS2*, are seen in 18–35% of small bowel adenocarcinomas including DAs [8–10]. MSI along with *KRAS* mutations represent the most common molecular defects in DA [7, 11, 12]. MSI is associated with prognosis in patients with colorectal cancer. Its prognostic value in DAs is worth investigation. *MGMT* methylation seems to favor mutations in cancer-related genes (e.g. *TP53* and *KRAS*). Kim et al. previously showed the association between *MGMT* methylation and *KRAS* G-to-A transition in a group of patients with carcinomas of the extrahepatic bile ducts, ampulla of Vater, and duodenum [7]. Due to the small number of duodenal carcinomas in the previous study, this correlation still needs validation.

The aims of this study were to assess the methylation status of *MGMT* gene in the largest series of stage III DAs reported to date and to establish whether or not methylation of *MGMT* might have prognostic or predictive value in patients with stage III DA.

## Material and Methods

### Study population

This retrospective cohort study included patients with pathologically confirmed DA who had a surgical resection. Patients were identified from the Johns Hopkins Hospital Oncology Clinical Information System from January 1997 to December 2009 and 155 duodenal adenocarcinomas patients who underwent surgical resection at our institution were identified. Patients who underwent preoperative chemotherapy/radiotherapy, lacked follow-up information or had missing archival primary tumors or corresponding matched normal samples were excluded. Formalin-fixed, paraffin-embedded (FFPE) tissue blocks of primary tumors and corresponding matched normal samples were collected from 107 patients. Tissue sections from the blocks were then reviewed by an expert gastrointestinal pathologist. After excluding ampullary tumors and low tumor cellularity sections, the remaining 64 stage III cases formed the final study cohort (Table 1). Ascertainment of survival was performed by using the Johns Hopkins electronic health records, the Cancer Registry and mortality was confirmed also within the Social Security Death Index. The Johns Hopkins Hospital Institutional Review Board approved this research protocol.

**Table 1:** Clinicopathological and molecular characteristics of patients and tumors by *MGMT* methylation status.

Characteristic	All patients (n = 64)	*MGMT*-U (n = 47)	*MGMT*-M (n = 17)	*P* ^a^
**Chemotherapy/radiotherapy**				0.756
No	17 (26.6%)	12 (25.5%)	5 (29.4%)	
Yes	47 (73.4%)	35 (74.5%)	12 (70.6%)	
**Sex**				0.602
Male	38 (59.4%)	27 (57.4%)	11 (64.7%)	
Female	26 (40.6%)	20 (42.6%)	6 (35.3%)	
**Age at surgery**				0.144^b^
< 60	21 (32.8%)	18 (38.3%)	3 (17.6%)	
≥ 60	43 (67.2%)	29 (61.7%)	14 (82.4%)	
**Tumor differentiation**				0.396
Well/moderate	32 (50.0%)	25 (53.2%)	7 (41.2%)	
Poor	32 (50.0%)	22 (46.8%)	10 (58.8%)	
**CIMP**				0.111
CIMP-	47 (73.4%)	37 (78.7%)	10 (58.8%)	
CIMP+	17 (26.6%)	10 (21.3%)	7 (41.2%)	
**MSI status**				1.000^b^
MSS	49 (76.6%)	36 (76.6%)	13 (76.5%)	
MSI	15 (23.4%)	11 (23.4%)	4 (23.5%)	
***KRAS***				0.430
Wild-type	39 (60.9%)	30 (63.8%)	9 (52.9%)	
Mutated	32 (32.3%)	17 (36.2%)	8 (47.1%)	

^a^*MGMT*-U versus *MGMT*-M, *χ*^2^ test unless indicated otherwise

^b^Fisher’s exact test.

Abbreviations: CIMP, CpG island methylator phenotype; MSS, microsatellite stable; MSI, microsatellite instability; U, unmethylated; M, methylated.

### Analyses of *KRAS* mutations, and microsatellite instability

Genomic DNA was extracted from FFPE tissues. Polymerase chain reaction (PCR) and sequencing targeted for *KRAS* codons 12 and 13 were performed [11, 13].

MSI status was determined using D2S123, D5S346, D17S250, BAT25, and BAT26 [14]. Microsatellite sizes were compared with those of normal adjacent tissue, and tumors with 2 or more of the markers exhibiting instability were classified as MSI-high. Tumors with only one marker exhibiting instability or no markers with instability were classified as MSI-low or microsatellite stable (MSS), respectively.

### Bisulfite modification and methylation analysis

Purified DNA (2 μg) was bisulfite treated and purified using the EZ DNA methylation kit (Zymo Research, Orange, CA) according to the manufacturer's instructions.

A 5-gene signature was used to assess the CpG island methylator phenotype (CIMP) status of the primary tumor tissue: *CACNA1G*, *IGF2*, *NEUROG1*, *RUNX3*, and *SOCS1* [15]. Methylation of these five genes and *MGMT* was quantified by MethyLight, a methylation-specific, probe-based, real-time PCR technique [12, 15, 16]. Alu was used as a normalization control reaction. All CIMP probes utilized a 5’ FAM fluorophore, a 3’ IBFQ quencher, and an internal ZEN quencher (Integrated DNA Technologies, Coraville, IA). DNA methylation was reported as the percent of methylated reference (PMR) = 100 × ((methylated reaction/Alu)_sample_/(methylated reaction/Alu)_M.SssI-reference_) [15]. We classified each marker as methylated when PMR ≥4. The PMR cut-off levels were set at plus two standard deviations of the average methylation levels observed in normal duodenal mucosa controls. Samples were considered CIMP+ if at least 3 out of the five studied genes were methylated [15].

### Statistical methods

Differences in categorical variables between study groups were analyzed using *χ*^2^ test or Fisher’s exact test. The primary end point for the study was disease-free survival (DFS), defined as the time from surgery to death or recurrence of disease, whichever occurred first. Overall survival (OS) was the secondary end point. Patients without evidence of death or recurrence were censored at last follow-up. Survival was estimated by using the Kaplan-Meier method and log-rank statistics computed to test for differences between survival curves for various prognostic factors. Univariate and multivariate Cox proportional hazard regression models included *MGMT* methylation, sex, age, tumor differentiation, R0 resection, chemoradiation, CIMP, MSI status, and *KRAS* mutations. Results of Cox regression are reported as hazard ratio (HR) with corresponding 95% confidence intervals (CI). All hypotheses tests were two-sided, and results were considered statistically significant for *P* values < 0.05. All calculations were performed using SPSS 16.0 software (SPSS Inc, Chicago, IL).

## Results

### Clinicopathologic characteristics and association with *MGMT* methylation or MSI status

DNA extraction, *MGMT* methylation testing by MethyLight, and MSI status testing were successful in all 64 patients. Seventeen patients (26.6%) out of the 64 patients tested were *MGMT*-methylated (*MGMT*-M, Table 1). Fifteen patients (23.4%) displayed MSI-high; 9 patients (14.1%) were MSI-low and 40 patients (62.5%) were MSS. Because extensive data indicate that tumors with MSI-low are biologically similar to those exhibiting MSS, both tumors were grouped together and henceforth are referred to as MSS in this study. Among the 17 (26.6%) patients demonstrating the CIMP positive (CIMP+), 7 (41.2%) were *MGMT*-M as well (Table 1). No correlation between CIMP and *MGMT* methylation status was observed (*P* = 0.111, Table 1).

Median age at diagnosis of DAs was 64.5 years (64.2 ± 14.3; mean ± SD). *MGMT*-unmethylated (*MGMT*-U) and *MGMT*-M subgroups showed no differences by gender, age, tumor differentiation, CIMP, MSI and *KRAS* mutation status or the receipt of chemotherapy/radiotherapy between the two groups (Table 1).

### *MGMT* methylation status as a prognostic marker

**Fig 1:**
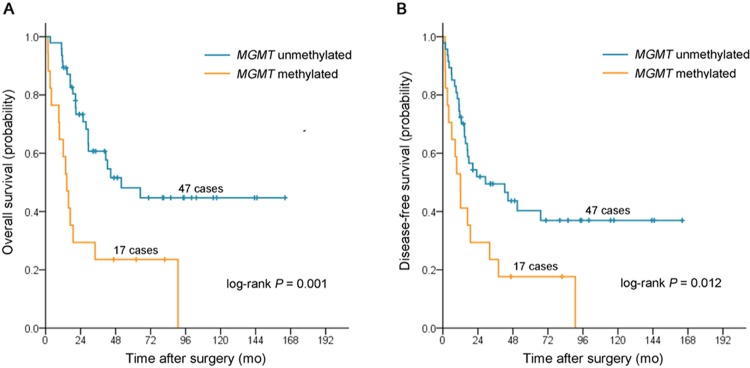
Kaplan-Meier survival estimates between patients with stage III duodenal adenocarcinomas with *MGMT* methylated and those with *MGMT* unmethylated. (**A**) overall survival, (**B**) disease-free survival.

The mean (SD) follow-up was 42.9 (28.5) months. There were 36 deaths, 24 recurrences, and 42 progressions at the end of follow-up. The median OS was 41.2 months (95% CI, 25.2 to 57.2 months), and the median DFS was 18.8 months (95% CI, 5.6 to 32.1 months). In Kaplan-Meier analysis of all patients, *MGMT*-M was associated with worse OS (log-rank *P* = 0.001; Fig 1A) and DFS (log-rank *P* = 0.012; Fig 1B). The median OS was 51.9 months (95% CI, 22.5 to 81.3 months) vs. 14.5 months (95% CI, 9.7 to 19.3 months), and the median DFS was 29.2 months (95% CI, 0 to 59.7 months) vs. 12.0 months (95% CI, 7.0 to 17.0 months) for patients with *MGMT*-U tumor vs. *MGMT*-M tumor, respectively. In univariate models, *MGMT*-M was associated with worse OS (HR, 3.01; 95% CI, 1.53 to 5.91; *P* = 0.001) and DFS (HR, 2.21; 95% CI, 1.17 to 4.17; *P* = 0.014). This remained statistically significant in multivariate models for OS (HR, 4.25; 95% CI, 2.00 to 9.05; *P* = 0.000) and for DFS (HR, 2.80; 95% CI, 1.43 to 5.48; *P* = 0.003; Table 2).

**Table 2:** Univariate and multivariate Cox proportional hazard analysis of overall survival (OS) and disease-free survival (DFS).

Characteristic	Total n	OS				DFS			
		Univariate		Multivariate		Univariate		Multivariate	
		HR (95% CI)	*P* value	HR (95% CI)	*P* value	HR (95% CI)	*P* value	HR (95% CI)	*P* value
***MGMT***									
U	47	1.00 (Referent)							
M	17	3.01 (1.53, 5.91)	0.001	4.25 (2.00, 9.05)	0.000	2.21 (1.17, 4.17)	0.014	2.80 (1.43, 5.48)	0.003
**Sex**									
Male	38	1.00 (Referent)							
Female	26	1.44 (0.75, 2.78)	0.275	1.62 (0.79, 3.35)	0.190	0.98 (0.53, 1.82)	0.950		
**Age**									
≥60	43	1.00 (Referent)							
<60	21	0.56 (0.26, 1.19)	0.131	0.57 (0.26, 1.27)	0.168	0.79 (0.41, 1.53)	0.488		
**Differentiation**									
Well/moderately	32								
Poorly	32	1.21 (0.63, 2.33)	0.568			1.54 (0.84, 2.84)	0.163	1.43 (0.77, 2.66)	0.260
**R0 resection**									
Yes	56								
No	8	1.16 (0.45, 2.98)	0.761			1.11 (0.47, 2.65)	0.807		
**Chemoradiation**									
Yes	47								
No	17	1.13 (0.51, 2.51)	0.759			0.85 (0.41, 1.73)	0.648		
**CIMP**									
CIMP-	47								
CIMP+	17	1.61 (0.80, 3.22)	0.180	2.84 (1.28, 6.32)	0.011	1.37 (0.70, 2.68)	0.361		
**MSI status**									
MSS	49								
MSI	15	0.43 (0.18, 1.04)	0.060	0.18 (0.06, 0.50)	0.001	0.35 (0.15, 0.84)	0.018	0.26 (0.10, 0.64)	0.003
***KRAS mutations***									
Absent	39								
Present	25	0.78 (0.40, 1.55)	0.482			0.87 (0.47, 1.63)	0.666		

Abbreviations: OS, overall survival; DFS, disease-free survival; HR, hazard ratio; CI, confidence interval; M, methylated; U, unmethylated; CIMP, CpG island methylator phenotype; MSS, microsatellite stable; MSI, microsatellite instability. A backward elimination with threshold of *P* = 0.300 was used to select variables in the final models

### Adjuvant treatment

Adjuvant treatment with fluorouracil-based chemotherapy/radiotherapy was administered in 47 patients, while 17 patients were treated with surgery alone. There was no significant improvement in OS for patients treated with adjuvant therapy when compared with patients who were not treated (HR, 1.13; 95% CI, 0.51 to 2.51; *P* = 0.759). When comparing DFS, there was no difference based on adjuvant treatment (HR, 0.85; 95% CI, 0.41 to 1.73; *P* = 0.648; Table 2).

**Fig 2:**
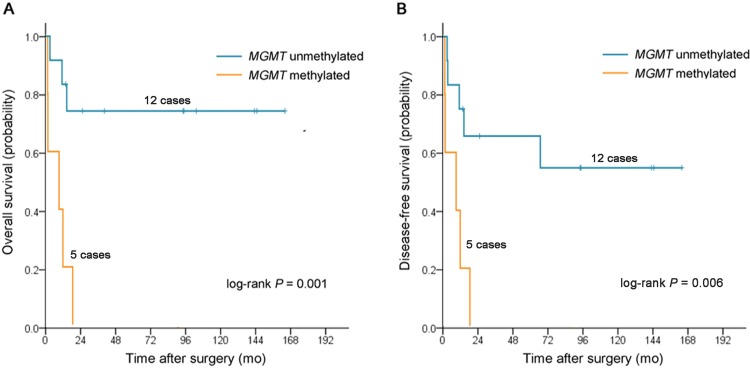
Kaplan-Meier survival estimates between patients with stage III duodenal adenocarcinomas with *MGMT* methylated and those with *MGMT* unmethylated in group treated with surgery alone. (**A**) overall survival, (**B**) disease-free survival.

**Fig 3:**
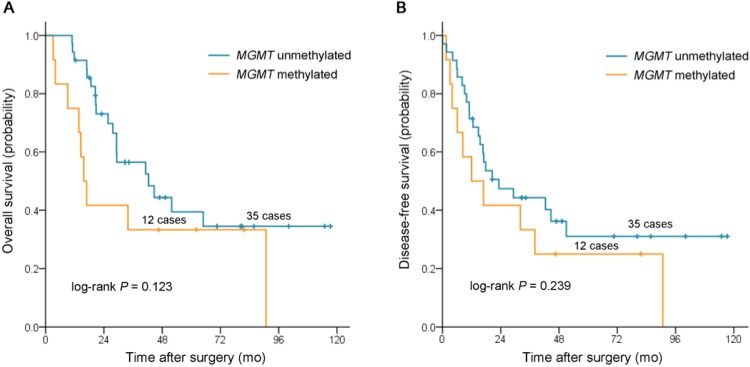
Kaplan-Meier survival estimates between patients with stage III duodenal adenocarcinomas with *MGMT* methylated and those with *MGMT* unmethylated in group treated with fluorouracil-based chemotherapy/radiotherapy. (**A**) overall survival, (**B**) disease-free survival.

In patients treated with surgery alone (n = 17), *MGMT*-M was associated with worse OS (HR, 7.88; 95% CI, 1.83 to 34.00; *P* = 0.006) and DFS (HR, 5.33; 95% CI, 1.40 to 20.30; *P* = 0.014) on univariate analysis. This remained statistically significant in multivariate models for OS (HR, 7.49; 95% CI, 1.04 to 53.84; *P* = 0.045) and OS (HR, 4.11; 95% CI, 1.03 to 16.40; *P* = 0.046). However, no association was observed between *MGMT* methylation status and both OS (HR, 1.85; 95% CI, 0.84 to 4.11; *P* = 0.130) and DFS (HR, 1.56; 95% CI, 0.74 to 3.30; *P* = 0.243; Table 3) in patients treated with chemotherapy/radiotherapy. In Kaplan–Meier analysis, there were also significant differences in survival time distributions between patients with *MGMT*-M and those with *MGMT*-U in the group treated with surgery alone (log-rank *P* = 0.001 for OS, Fig 2A; log-rank = 0.006 for DFS, Fig 2B). The median OS was not reached vs. 9.4 months (95% CI, 0 to 25.7 months), and the median DFS was not reached vs. 9.4 months (95% CI, 0 to 25.7 months) for patients with *MGMT*-U tumor vs. *MGMT*-M tumor, respectively. No significant differences were found between patients with *MGMT*-M tumor and those with *MGMT*-U tumor in the group treated with chemotherapy/radiotherapy (log-rank *P* = 0.123 for OS, Fig 3A; log-rank = 0.239 for DFS, Fig 3B).

**Table 3:** Univariate and multivariate Cox proportional hazard analysis of overall survival (OS) and disease-free survival (DFS) by *MGMT* methylation and chemotherapy/radiotherapy treatment status.

Characteristic	Total n	OS				DFS			
		Univariate		Multivariate		Univariate		Multivariate	
		HR (95% CI)	*P* value	HR (95% CI)	*P* value	HR (95% CI)	*P* value	HR (95% CI)	*P* value
**Untreated**									
*MGMT*-U	12								
*MGMT*-M	5	7.88 (1.83, 34.00)	0.006	7.49 (1.04, 53.84)	0.045	5.33 (1.40, 20.30)	0.014	4.11 (1.03, 16.40)	0.046
**Treated**									
*MGMT*-U	35								
*MGMT*-M	12	1.85 (0.84, 4.11)	0.130			1.56 (0.74, 3.30)	0.243		

Abbreviations: OS, overall survival; DFS, disease-free survival; HR, hazard ratio; CI, confidence interval; *MGMT*-M, *MGMT*-methylated; *MGMT*-U, *MGMT*-unmethylated

## Discussion

The present study was designed to better understand the contribution of methylation of *MGMT* for patients with stage III DAs and to determine its effect in response to fluorouracil-based adjuvant chemotherapy/radiotherapy in a cohort of patients. Our results indicate that, *MGMT* methylation is a reliable and independent prognostic factor in DAs. *MGMT* methylation is associated with poor prognosis in patients with stage III DAs. It seems that fluorouracil-based chemotherapy/radiotherapy does not improve outcomes in patients with stage III DAs. However, in the subsets of DAs with *MGMT* methylation fluorouracil-based chemotherapy/radiotherapy may confer a survival benefit.

*MGMT* methylation has been associated with various cancers. Specifically, *MGMT* methylation was seen in 39–53% of CRCs [17, 18], 11% of gastric cancer [19], 30–38% of lung cancer [20, 21], 34–72% of esophageal cancer [22], 34% of soft tissue sarcomas [23], 58% of breast cancer [24], and 30–70% of glioblastoma [25, 26]. In this study, we analyzed a large cohort of patients with stage III DAs and showed that *MGMT* methylation existed in 26.6% (17/64) of the tumors.

It was reported that inactivation of *MGMT* by promoter methylation was theoretically associated with the presence of *KRAS* G>A transitions in CRC [27]. Their data suggested that epigenetic silencing of *MGMT* by methylation was strongly associated with, and preceded, G>A mutations in *KRAS* in colorectal tumorigenesis. Some studies proved this possible association in CRCs [28, 29], however, we did not find this link between these two events in DAs (*P* = 0.226; data not shown). This can be secondary to various causes including methodology issues (type of methylation assay, small sample size, intratumor heterogeneity) and most importantly, alternative molecular mechanisms that cause DAs. The concurrence of these epigenetic and genetic lesions in different tumors suggests a more complex relationship between these events. For example, *MGMT* methylation is common [26], but *KRAS* mutations are relatively rare in glioblastoma [30]. Nagy et al. also showed that no conclusions could be drawn with regard to mutation type and methylation in endometrial cancers [31]. In a study of 62 gastric cancer tissue samples, *KRAS* mutations were detected in only one (1.6%) sample and *MGMT* methylation was detected in 13 (21%) samples, and no connection was shown between *KRAS* mutations and *MGMT* methylation [32]. Similar results were shown in a study of 62 soft tissue sarcomas with *MGMT* methylation 33.9% (21/62) and *KRAS* mutations 3.7% (2/62) [23]. In a large cohort study with 1123 CRC, a strong association with *MGMT* methylation was found with *KRAS* mutations both in univariate analysis (OR 2.3, 95% CI 1.7–3.0, *P* < 0.0001) and multivariate analysis (OR 1.9, 95% CI 1.5–2.6, *P* < 0.0001). But on classification of the *KRAS* mutant cancers by mutation type, no association was found between *MGMT* methylation and G>A mutations compared with non-G>A mutations, and in fact frequency of *MGMT*-M and *MGMT*-U tumors was approximately equal for each mutation category [33].

In previous studies, the significance of the correlation between *MGMT* methylation and prognosis of patients was controversial [21, 25, 34–36]. In present study, the impact of *MGMT* methylation on patient survival was assessed by univariate and multivariate analyses. Cox proportional hazard models indicated that methylation of *MGMT* was strongly associated with poor survival in DAs patients.

Despite the absence of prospective randomized data clarifying the role of adjuvant therapy in DAs, the use of adjuvant therapy has increased. Data from the National Cancer Database shows a spread use of adjuvant chemoradiation in small bowel cancers (including 49.1%-58.8% DAs) from 8.1% in 1985 to 22.2% in 2005 (*P* < 0.0001) [37]. In all likelihood, this trend reflects the poor outcome of high-risk dissected DAs, the known efficacy of systemic chemoradiation in the metastatic setting and the significant survival benefit of adjuvant therapy in patients with CRC.

Several studies have individually examined the results of adjuvant therapy after resection of DA. In 1980, Alwmark et al. suggested that chemoradiation might improve the survival of patients with DA [1]. Since then, advances in chemotherapy and radiotherapy have developed, but chemoradiation has commonly been reserved for palliation of DAs. Our institution has previously published a pilot study on 14 patients with node-positive DA who underwent pancreaticoduodenectomy followed by adjuvant fluorouracil-based chemoradiation [38]. This study suggested that adjuvant chemoradiation contributed improved local control compared with historical controls treated with surgery alone (93% vs. 67%), but did not lengthen overall survival (5 year, 44% vs. 43%). However, in this follow up study from our institution of a larger cohort of patients we were unable to reproduce this positive effect of chemoradiation for either local control or OS [39]. Another retrospective study of 103 patients with DA (including 46 stage III DAs) from Massachusetts General Hospital compared patients who underwent resection alone with those who received resection and adjuvant and/or neoadjuvant chemotherapy/chemoradiation and found no marked improvement in OS, or time to recurrence [6]. A similar study of 32 patients with DA from Duke University Medical Center also failed to show a beneficial effect of adjuvant chemoradiation both in terms of OS (44% vs. 57%), disease-free survival (44% vs. 54%) or local control (49% vs. 70%) [40]. In an analysis of 1,611 cases on long-term outcome after resection of DA by utilizing the Surveillance, Epidemiology, and End Results (SEER) database, a large population-based cancer registry showed that the use of radiation was associated with improvements in survival on univariate analysis, but this effect disappeared after controlling for other variable [41].

In this study, we showed that patients treated with adjuvant therapy had similar prognosis to those treated with surgery alone. In patients treated with surgery alone, patients with *MGMT*-M tumor had worse OS and DFS compared with those with *MGMT*-U tumor. However, in patients undergoing adjuvant fluorouracil-based chemotherapy/radiotherapy, outcomes became comparable between patients with *MGMT*-M tumor and those with *MGMT*-U tumor. This might be, to some extent, due to differential responses to chemotherapy/radiotherapy between these two subtypes of tumor. Nevertheless, this phenomenon deserves further investigation. The finding is potentially of great significance, as the addition of adjuvant chemotherapy/radiotherapy in DAs is currently a matter of great debate.

Alkylating agent temozolomide is now the chemotherapeutic agent most regularly used in patients with newly diagnosed glioblastoma. It is well established that *MGMT* methylation is a promising predictor of prolonged prognosis in patients with glioblastoma receiving temozolomide [42, 43]. In a pivotal randomized trial investigating the value of temozolomide added to radiotherapy in patients with glioblastoma, median survival in patients with methylated *MGMT* promoter increased from 15.3 months (95% CI 13.0–20.9) with radiotherapy alone to 21.7 months (17.4–30.4) with radiotherapy and temozolomide (hazard ratio [HR] 0.51, 95% CI 0.31–0.84). However, patients with unmethylated *MGMT* promoter in the tumor showed only a marginal benefit from addition of temozolomide, with a median survival of 12.7 months (95% CI 11.6–14.4) compared with 11.8 months (9.7–14.1) for patients treated with radiotherapy alone (HR 0.69, 95% CI 0.47–1.02) [44]. However, the value of *MGMT* methylation as a prognostic or predictive marker for patients treated with other specific regimens of anticancer agents remains a matter of debate to date. A previous study has shown that CRC patients who received oral fluorouracil-based adjuvant chemotherapy had a low recurrence rate when the tumor revealed methylation in its *MGMT* promoter [45]. Their in vitro study also proved an enhancement of fluorouracil anti-tumor effect for CRC and other malignancies with *MGMT* methylation by controlling the levels of MGMT in tumor [46]. It was hypothesized that tumor cells with methylation of *MGMT* are likely to remain in G2/M checkpoint, resulting in increased sensitivity to chemoradiation [47, 48].

Our results show that *MGMT* methylation is an important prognostic factor in stage III DAs. Our data also suggest a possible role for fluorouracil-based chemotherapy/radiotherapy in management of stage III DAs patients with *MGMT* methylation and *MGMT*-M may also then have a predictive role. Further studies in larger samples will help validate these.

## Supporting Information

S1 File SPSS file for statistical analysis. (SAV)
